# Gait Analysis After Reverse Total Shoulder Arthroplasty

**DOI:** 10.2106/JBJS.OA.26.00015

**Published:** 2026-06-03

**Authors:** Takahiro Maeda, Hiroyasu Ikegami, Shu Yoshizawa, Hideaki Ishii, Misato Sakamoto, Tomoyasu Homma, Osahiko Tsuji, Daiki Higashi, Shouta Shimoyama

**Affiliations:** 1 Department of Orthopedic Surgery, Toho University Medical Center, Ohashi Hospital, Tokyo, Japan; 2 Division of Physical Therapy, Department of Rehabilitation, Toho University Medical Center, Ohashi Hospital, Tokyo, Japan

## Abstract

**Background:**

Shoulder pain in older adults is associated with reduced mobility and impaired quality of life. Reverse total shoulder arthroplasty (RTSA) reliably improves shoulder pain and function; however, its effect on whole-body function, particularly gait and trunk stability, has not been fully elucidated. We aimed to investigate longitudinal changes in gait performance and trunk stability following RTSA using triaxial accelerometer-based gait analysis.

**Methods:**

This retrospective observational case series included 32 patients (3 male and 29 female; mean age, 77.5 ± 8.1 years) who underwent RTSA and gait analysis preoperatively and postoperatively at 1 week, 1 month, and/or 3 months. Gait analysis was performed under normal and maximum-speed walking conditions using a triaxial accelerometer attached to the third lumbar vertebra. Temporal and spatial gait parameters and trunk acceleration-based indices, including root mean square (RMS), velocity-corrected RMS (RMS*), and the symmetry index (SI), were evaluated. Paired *t*-tests and linear mixed-effects models were used for statistical analyses.

**Results:**

During normal walking, trunk acceleration indices (RMS in all axes, resultant RMS, and RMS*) significantly decreased from 1 month postoperatively, whereas walking time significantly decreased and stride length increased from 1 month postoperatively, indicating early improvement in temporal and spatial gait parameters. During maximum-speed walking, significant reductions in trunk acceleration indices and improvements in the SI were observed at 3 months postoperatively. Linear mixed-effects model analysis demonstrated significant postoperative improvements in walking speed, stride length, walking time, walking cycle, and trunk stability indices. Improvements in gait stability preceded or paralleled improvements in gait efficiency.

**Conclusions:**

RTSA was associated with reductions in trunk acceleration variability (RMS and RMS*) and improvements in gait efficiency, with changes emerging by 1 month during normal walking and by 3 months during maximum-speed walking. These findings suggest that RTSA may contribute to improved whole-body gait control in addition to restoring shoulder function.

**Level of Evidence:**

Level IV, therapeutic case series. See Instructions for Authors for a complete description of levels of evidence.

## Introduction

Shoulder pain is common in older adults and has been associated with reduced walking ability and poorer physical quality of life^1^. Reverse total shoulder arthroplasty (RTSA) is increasingly performed for cuff tear arthropathy and glenohumeral osteoarthritis because it can restore shoulder elevation by deltoid compensation even when rotator cuff function is deficient. Although RTSA reliably improves pain and shoulder function, its potential effect on whole-body function—particularly gait—has not been quantified. We hypothesized that postoperative pain relief and improved shoulder mobility would be accompanied by measurable improvements in gait performance and trunk stability. Therefore, we performed serial gait analyses before and after RTSA using a triaxial accelerometer. To our knowledge, this is the first study to evaluate gait parameters after shoulder arthroplasty.

## Materials and Methods

This study included 32 patients (3 male and 29 female) who underwent RTSA at our institution between August 2022 and August 2024 and completed gait analysis preoperatively and at 1 week and 1 and 3 months postoperatively. These time points reflect the standard clinical follow-up schedule at our institution, with assessments performed at 1 week during hospitalization and at 1 and 3 months during routine outpatient visits. All patients were Japanese. Patients who were unable to complete gait analysis or who had severe lower-limb or neurological disorders affecting gait were excluded. All patients were able to ambulate independently without assistive devices at each evaluation point. No patients opted out of the study. The study protocol was approved by the Ethics Committee of Toho University Ohashi Medical Center (approval number T2024-3171) and was conducted in accordance with the tenets of the Declaration of Helsinki. Information about the study was disclosed on the institutional website, providing participants with the opportunity to opt out.

The mean age at surgery was 77.5 ± 8.1 (range, 60-91) years. No postoperative complications occurred. All patients ambulated independently without walking aids. Indications for surgery were cuff tear arthropathy and glenohumeral osteoarthritis. All procedures were performed by a single surgeon.

RTSA was performed using a deltopectoral approach. After detachment of the subscapularis, the implant was placed with navigation assistance using the Exactech Equinoxe System (Exactech). The long head of the biceps tendon was tenodesed, and the subscapularis was repaired. Postoperative rehabilitation began on postoperative day 1 with pendulum exercises, without external immobilization.

Gait analysis was conducted using a triaxial accelerometer (AYUMI EYE; Waseda EHA). The sensor (18.5 g; 62.4 × 30.9 × 11.8 mm) recorded acceleration at 31.25 Hz with a ±4 G detection range and 8 mG/digit sensitivity. Data were transmitted wirelessly through Bluetooth. The sensor was attached to the spinous process of the third lumbar vertebra.

Patients walked on level ground under normal and maximum-speed conditions. Temporal and spatial parameters included walking time, gait velocity, stride length, and walking cycle. Trunk stability was assessed using the resultant root mean square (RMS) of trunk acceleration, as well as axis-specific RMS values (mediolateral, anteroposterior, and vertical), velocity-corrected RMS (RMS*), walking cycle variability, average grounding time, grounding time ratio, and the symmetry index (SI).

### RMS

Trunk acceleration was recorded along 3 orthogonal axes: mediolateral (X), vertical (Y), and anteroposterior (Z). For each axis, the RMS of acceleration was calculated as^2^:

$$RMS=\sqrt{\frac{1}{N}\overset{N}{\underset{i=1}{\sum }}{\left(a_{i}-\bar{a}\right)}^{2}}$$

where $a_{i}$ is the instantaneous acceleration at time *i*, $\bar{a}$ is the mean acceleration over one gait cycle, and *N* is the number of data points.

The resultant RMS (resRMS), representing the overall magnitude of trunk acceleration, was calculated as the square root of the sum of squared RMS values from the 3 axes^2,3^:

$$resRMS=\sqrt{\left(RM{S_{x}}^{2}+RMS{ᵧ}^{2}+RMS_{z}^{2}\right)}$$

Higher RMS values indicate greater trunk motion and reduced gait stability. RMS reflects trunk sway during walking, with lower values indicating greater stability.

### Velocity-Corrected RMS (RMS*)

To evaluate trunk stability independent of walking speed, resRMS was normalized by gait velocity to obtain the velocity-corrected RMS (RMS*)^3,4^:

$$RMS^{*}=\frac{RMS}{V}$$

where *V* is walking velocity (m/s). Lower RMS* values indicate better trunk control. RMS* adjusts trunk acceleration for walking speed, allowing comparison of trunk stability independent of gait velocity.

RMS* was calculated for each participant as the resultant RMS divided by gait velocity, and the resulting values were then summarized.

### SI

The SI was calculated to evaluate left–right gait symmetry using the following formula^5^:

$$SI\left(\%\right)=\frac{\left|L-R\right|}{\left(L+R\right)/2}\times 100$$

where *L* and *R* represent mean stance times. Lower SIs indicate greater gait symmetry. Clinically, a lower SI reflects more balanced left–right loading and coordinated gait patterns.

EZR (ver. 1.61) and R (ver. 4.3.1) were used for statistical analysis. A paired *t*-test was used for comparisons between time points. To analyze missing data, we used a linear mixed-effects model (LMM), with measurement time (preoperative, 1 week, 1 month, and 3 months) as a fixed effect and individual differences as a random effect. This longitudinal mixed-effects approach evaluates within-subject changes over time while accounting for interindividual variability.

The level of statistical significance was set at p < 0.05.

## Results

The data of 32 patients (3 male and 29 female; mean age, 77.5 ± 8.2 years) who underwent RTSA were analyzed. Longitudinal analyses were performed using an LMM, and paired *t*-tests were conducted for complete datasets. Overall, 16 patients were included in the preoperative-1-week comparison, 28 in the preoperative-1-month comparison, and 19 in the preoperative 3-month comparison.

### Normal Walking (Paired *t*-test)

No significant changes were observed at 1 week. From 1 month onward, walking time decreased, stride length increased, and walking cycle shortened (Table I). The trunk acceleration indices (RMS in all axes, resultant RMS, and RMS*) significantly decreased at 1 and 3 months. The SI showed an improving trend but did not reach statistical significance.

**TABLE I:** Comparison of Gait Parameters Under Normal Walking Condition Before and After RTSA (Paired *t*-test)

Normal Walking	Pre	1W	p (Pre–1W)	1M	p (Pre–1M)	3M	p (Pre–3M)
Gait velocity (m/s)	0.985 ± 0.132	0.992 ± 0.141	0.597	1.013 ± 0.147	0.078	1.074 ± 0.163	0.053
Stride length (m)	1.008 ± 0.118	1.012 ± 0.127	0.87	1.034 ± 0.123	0.055	1.059 ± 0.131	0.038*
Walking time (s)	7.024 ± 1.582	7.081 ± 1.541	0.64	6.804 ± 1.449	0.032*	6.610 ± 1.405	0.001**
Walking cycle (s)	1.053 ± 0.082	1.069 ± 0.085	0.233	1.045 ± 0.079	0.063	1.033 ± 0.072	0.050*
RMS *x*-axis	0.158 ± 0.027	0.156 ± 0.025	0.606	0.151 ± 0.024	0.043*	0.148 ± 0.023	0.012*
RMS *y*-axis	1.432 ± 0.154	1.401 ± 0.158	0.105	1.312 ± 0.143	0.012*	1.211 ± 0.134	0.009**
RMS *z*-axis	0.439 ± 0.064	0.433 ± 0.059	0.217	0.414 ± 0.058	0.011*	0.398 ± 0.054	0.007**
Walking cycle variability	0.021 ± 0.006	0.022 ± 0.005	0.040*	0.020 ± 0.005	0.063	0.019 ± 0.004	0.059
Grounding time average (s)	0.641 ± 0.042	0.646 ± 0.040	0.252	0.633 ± 0.041	0.058	0.627 ± 0.037	0.05
Grounding time ratio (%)	60.5 ± 4.1	60.7 ± 4.2	0.786	59.9 ± 4.0	0.087	59.6 ± 3.9	0.057
Resultant RMS	1.472 ± 0.182	1.469 ± 0.177	0.872	1.393 ± 0.169	0.035*	1.303 ± 0.157	0.009**
RMS*	1.381 ± 0.164	1.374 ± 0.162	0.26	1.311 ± 0.154	0.041*	1.256 ± 0.148	0.011*
SI	1.027 ± 0.078	1.025 ± 0.079	0.178	1.018 ± 0.076	0.067	1.011 ± 0.074	0.055

pre = preoperative, RMS = root mean square trunk acceleration, RMS* = velocity-corrected RMS, RTSA = reverse total shoulder arthroplasty, and SI = symmetry index.

Values are presented as means ± SDs. p-values were obtained using paired *t*-tests comparing each postoperative time point (1 week, 1 month, and 3 months) with the preoperative baseline. Preoperative values are presented as descriptive statistics for the entire cohort (n = 32). Paired *t*-tests were conducted using subsets of patients with complete paired data for each comparison (pre vs. 1 week: n = 16; pre vs. 1 month: n = 28; pre vs. 3 months: n = 19).

* p < 0.05.

** p < 0.01.

### Maximum Walking (Paired *t*-test)

At 3 months postoperatively, stride length, walking time, and walking cycle improved significantly (Table II). The trunk acceleration indices (RMS in all axes, resultant RMS, and RMS*) significantly decreased, and the SI significantly improved.

**TABLE II:** Comparison of Gait Parameters Under Maximum Walking Condition Before and After RTSA (Paired *t*-test)

Maximum Walking	Pre	1W	p (Pre–1W)	1M	p (Pre–1M)	3M	p (Pre–3M)
Gait velocity (m/s)	1.204 ± 0.183	1.229 ± 0.186	0.185	1.247 ± 0.190	0.068	1.271 ± 0.193	0.051
Stride length (m)	1.187 ± 0.167	1.194 ± 0.171	0.269	1.216 ± 0.180	0.058	1.232 ± 0.189	0.045*
Walking time (s)	5.618 ± 1.280	5.591 ± 1.266	0.326	5.525 ± 1.232	0.042*	5.476 ± 1.208	0.026*
Walking cycle (s)	0.958 ± 0.081	0.969 ± 0.079	0.261	0.954 ± 0.077	0.054	0.947 ± 0.075	0.043*
RMS *x*-axis	0.161 ± 0.029	0.159 ± 0.028	0.222	0.156 ± 0.026	0.049*	0.153 ± 0.025	0.040*
RMS *y*-axis	1.521 ± 0.174	1.504 ± 0.179	0.843	1.442 ± 0.167	0.035*	1.401 ± 0.162	0.028*
RMS *z*-axis	0.471 ± 0.069	0.465 ± 0.067	0.079	0.447 ± 0.064	0.023*	0.431 ± 0.061	0.015*
Walking cycle variability	0.019 ± 0.005	0.020 ± 0.006	0.562	0.018 ± 0.005	0.075	0.018 ± 0.005	0.061
Grounding time average (s)	0.583 ± 0.039	0.586 ± 0.037	0.246	0.574 ± 0.038	0.051	0.567 ± 0.036	0.046*
Grounding time ratio (%)	57.9 ± 3.8	58.1 ± 3.9	0.597	57.3 ± 3.7	0.09	56.8 ± 3.6	0.068
Resultant RMS	1.559 ± 0.196	1.557 ± 0.192	0.958	1.476 ± 0.184	0.046*	1.412 ± 0.176	0.038*
RMS*	1.478 ± 0.179	1.472 ± 0.177	0.269	1.411 ± 0.169	0.043*	1.359 ± 0.162	0.029*
SI	1.036 ± 0.081	1.034 ± 0.083	0.086	1.028 ± 0.078	0.056	1.022 ± 0.076	0.049*

pre = preoperative, RMS = root mean square trunk acceleration, RMS* = velocity-corrected RMS, RTSA = reverse total shoulder arthroplasty, and SI = symmetry index.

Values are presented as means ± SDs. p-values were obtained using paired *t*-tests comparing each postoperative time point (1 week, 1 month, and 3 months) with the preoperative baseline. Preoperative values are presented as descriptive statistics for the entire cohort (n = 32). Paired *t*-tests were conducted using subsets of patients with complete paired data for each comparison (pre vs. 1 week: n = 16; pre vs. 1 month: n = 28; pre vs. 3 months: n = 19).

* p < 0.05.

### Normal Walking (LMM)

**Fig. 1:**
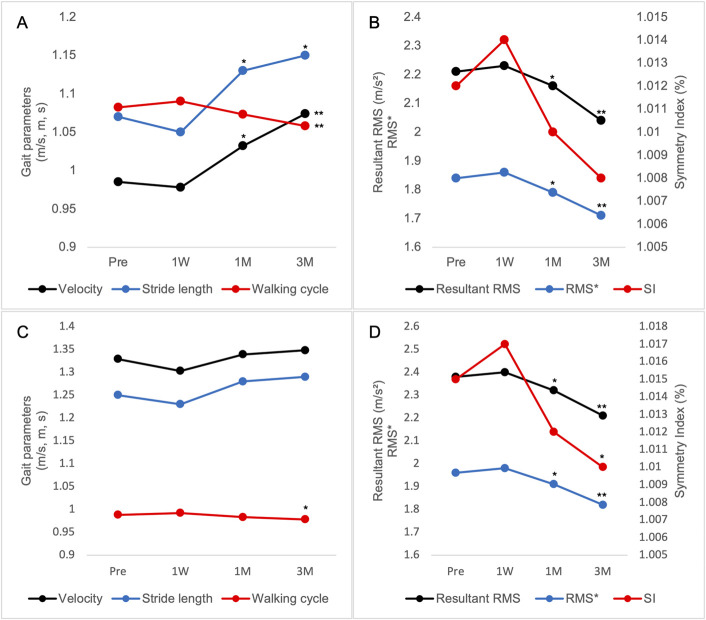
Longitudinal changes in gait parameters after RTSA. (**Fig. 1-A**) Gait efficiency during normal walking. (**Fig. 1-B**) Trunk stability during normal walking. (**Fig. 1-C**) Gait efficiency during maximum walking. (**Fig. 1-D**) Trunk stability during maximum walking. *p < 0.05; **p < 0.01 compared with the preoperative baseline, based on linear mixed-effects model analysis. RTSA = reverse total shoulder arthroplasty.

The LMM analysis demonstrated significant postoperative improvements in walking speed, stride length, walking time, and walking cycle (Table III and Fig. 1-A). The trunk acceleration indices (RMS in all axes, resultant RMS, and RMS*) significantly decreased, and the average grounding time shortened (Fig. 1-B). The SI showed a nonsignificant improving trend.

**TABLE III:** Changes in Gait Parameters Under Normal Walking Condition Before and After RTSA (LMM Analysis)

Normal Walking	Pre	1W	p (Pre–1W)	1M	p (Pre–1M)	3M	p (Pre–3M)
Gait velocity (m/s)	0.985 ± 0.152	0.978 ± 0.143	0.41	1.032 ± 0.139	0.038*	1.074 ± 0.128	0.004**
Stride length (m)	1.070 ± 0.140	1.050 ± 0.150	0.09	1.130 ± 0.140	0.038*	1.150 ± 0.130	0.011*
Walking time (s)	4.120 ± 0.570	4.250 ± 0.620	0.19	3.920 ± 0.480	0.011*	3.740 ± 0.450	0.001**
Walking cycle (s)	1.082 ± 0.083	1.090 ± 0.089	0.231	1.073 ± 0.080	0.079	1.058 ± 0.077	0.006**
RMS *x*-axis	1.204 ± 0.154	1.234 ± 0.167	0.22	1.185 ± 0.145	0.38	1.112 ± 0.136	0.012*
RMS *y*-axis	1.432 ± 0.183	1.389 ± 0.178	0.11	1.298 ± 0.159	0.009**	1.211 ± 0.148	<0.001**
RMS *z*-axis	1.315 ± 0.162	1.307 ± 0.153	0.68	1.257 ± 0.141	0.039*	1.198 ± 0.132	0.007**
Walking cycle variability	0.021 ± 0.004	0.022 ± 0.005	0.337	0.020 ± 0.004	0.262	0.019 ± 0.004	0.068
Grounding time average (s)	0.68 ± 0.09	0.70 ± 0.08	0.19	0.66 ± 0.07	0.15	0.64 ± 0.06	0.026*
Grounding time ratio (%)	60.3 ± 2.8	61.2 ± 3.0	0.24	59.8 ± 2.6	0.43	59.0 ± 2.4	0.11
Resultant RMS	2.21 ± 0.20	2.23 ± 0.21	0.51	2.16 ± 0.18	0.028*	2.04 ± 0.17	<0.001**
RMS*	1.84 ± 0.15	1.86 ± 0.16	0.49	1.79 ± 0.13	0.041*	1.71 ± 0.12	0.002**
SI	1.012 ± 0.031	1.014 ± 0.034	0.51	1.010 ± 0.029	0.35	1.008 ± 0.028	0.051

LMM = linear mixed model, pre = preoperative, RMS = root mean square trunk acceleration, RMS* = velocity-corrected RMS, RTSA = reverse total shoulder arthroplasty, and SI = symmetry index.

Values are presented as means ± SDs. p-values were obtained from linear mixed model analyses comparing each postoperative time point (1 week, 1 month, and 3 months) with the preoperative baseline.

Values represent model-estimated means derived from linear mixed-effects models.

* p < 0.05.

** p < 0.01.

### Maximum Walking (LMM)

Walking speed and stride length did not change significantly, whereas walking cycle significantly shortened (Table IV and Fig. 1-C). The trunk acceleration indices (RMS in all axes, resultant RMS, and RMS*) significantly decreased, and the SI significantly improved (Fig. 1-D).

**TABLE IV:** Changes in Gait Parameters Under Maximum Walking Condition Before and After RTSA (LMM Analysis)

Maximum Walking	Pre	1W	p (Pre–1W)	1M	p (Pre–1M)	3M	p (Pre–3M)
Gait velocity (m/s)	1.329 ± 0.162	1.303 ± 0.158	0.24	1.339 ± 0.155	0.61	1.348 ± 0.142	0.43
Stride length (m)	1.250 ± 0.160	1.230 ± 0.150	0.16	1.280 ± 0.140	0.12	1.290 ± 0.150	0.15
Walking time (s)	3.310 ± 0.440	3.380 ± 0.460	0.21	3.250 ± 0.410	0.18	3.220 ± 0.390	0.07
Walking cycle (s)	0.988 ± 0.081	0.992 ± 0.086	0.384	0.983 ± 0.081	0.312	0.978 ± 0.079	0.047*
RMS *x*-axis	1.312 ± 0.171	1.327 ± 0.164	0.41	1.282 ± 0.155	0.23	1.256 ± 0.142	0.034*
RMS *y*-axis	1.525 ± 0.195	1.488 ± 0.189	0.14	1.421 ± 0.176	0.015*	1.356 ± 0.162	0.004**
RMS *z*-axis	1.432 ± 0.175	1.421 ± 0.168	0.63	1.379 ± 0.161	0.045*	1.348 ± 0.151	0.013*
Walking cycle variability	0.019 ± 0.004	0.020 ± 0.005	0.3	0.018 ± 0.004	0.25	0.017 ± 0.004	0.06
Grounding time average (s)	0.62 ± 0.08	0.63 ± 0.08	0.28	0.61 ± 0.07	0.23	0.60 ± 0.07	0.051
Grounding time ratio (%)	58.4 ± 2.6	59.0 ± 2.8	0.35	57.9 ± 2.4	0.38	57.1 ± 2.2	0.043*
Resultant RMS	2.38 ± 0.23	2.40 ± 0.24	0.52	2.32 ± 0.21	0.043*	2.21 ± 0.19	<0.001**
RMS*	1.96 ± 0.16	1.98 ± 0.17	0.49	1.91 ± 0.15	0.041*	1.82 ± 0.13	0.002**
SI	1.015 ± 0.029	1.017 ± 0.031	0.49	1.012 ± 0.028	0.33	1.010 ± 0.027	0.042*

LMM = linear mixed model, pre = preoperative, RMS = root mean square of trunk acceleration, RMS* = velocity-corrected RMS, RTSA = reverse total shoulder arthroplasty, and SI = symmetry index.

Values are presented as means ± SDs. p-values were obtained from linear mixed model analyses comparing each postoperative time point (1 week, 1 month, and 3 months) with the preoperative baseline.

Values represent model-estimated means derived from linear mixed-effects models.

* p < 0.05.

** p < 0.01.

## Discussion

To our knowledge, this study is the first to analyze longitudinal gait dynamics after RTSA. Previous studies have focused on shoulder function, and few have examined its influence on whole-body gait stability.

Upper limb motion is an important component of postural control during gait. Previous studies have shown that arm swing contributes to recovery from perturbations and reduces energy expenditure through active muscular contraction and passive mechanisms^6-9^. These findings indicate that coordinated upper limb motion is integral to efficient and stable human gait.

In the early postoperative period (1 week), although paired *t*-tests showed slight increases in walking speed and stride length, the LMM analysis indicated a time-series trend of decrease from the preoperative period, resulting in differing findings depending on the evaluation method used. This early postoperative phase likely reflects transient surgical effects and residual pain, making consistent improvement difficult to interpret.

By contrast, RMS showed a decreasing trend from the early postoperative period. By 1 month after surgery, the RMS values along the *x*-, *y*-, and *z*-axes, as well as the resultant RMS and RMS*, had decreased significantly. A reduction in RMS reflects decreased trunk sway during walking^10^. This improvement is believed to reflect the resumption of upper limb swing as shoulder pain decreased and range of motion improved, resulting in more coordinated trunk and pelvic motion. Although reduced trunk acceleration is generally interpreted as improved stability, it may also reflect a more cautious or rigid gait strategy in older individuals. In this study, however, the concurrent improvements in walking speed and stride length suggest that the reduction in RMS was accompanied by enhanced gait efficiency, supporting the interpretation that dynamic stability improved.

At 3 months postoperatively, the temporal and spatial gait parameters showed clearer improvement. Walking speed significantly increased in the LMM analysis, whereas stride length showed significant increases in the paired *t*-test and LMM analysis. Walking time and gait cycle were also significantly shortened as early as 1 month postoperatively, indicating that an efficient gait pattern had been regained. These findings suggest steady postoperative improvement in gait function.

Regarding the SI, which reflects left–right gait symmetry, a trend toward improvement was observed during normal walking in both the paired *t*-test and LMM analysis; however, these changes did not reach statistical significance (p = 0.055 and 0.051, respectively). By contrast, a significant improvement was observed during maximum walking at 3 months postoperatively (p = 0.049 and 0.042, respectively). Gait symmetry is an important indicator of gait quality and stability^11^, and compensatory gait patterns that protect the affected side in patients with pain or functional impairment are known to increase asymmetry^12^. Particularly, an increase in the SI is considered indicative of worsening asymmetrical gait and pain-avoidance behavior^13^. The observed improvement in the SI suggests a reduction in compensatory asymmetrical gait following RTSA and recovery of neuromuscular function involved in gait control. Moreover, in older adults, gait symmetry is associated with balance function and fall risk^14^; therefore, improvements in the SI have clinical relevance not only for qualitative gait enhancement but also for fall prevention.

Under maximum walking conditions, although walking speed and stride length did not show significant differences in the LMM analysis, paired *t*-tests conducted at 3 months postoperatively revealed a significant increase in stride length (p = 0.045). In addition, RMS values along each axis, as well as resultant RMS and RMS*, decreased significantly at 3 months postoperatively, and SI improved. These findings indicate suppression of trunk sway and improved left–right coordination even under higher-load walking conditions, suggesting that gait stability after RTSA improves regardless of walking speed.

Taken together, these results demonstrate that RTSA not only reduces shoulder pain and improves range of motion but also enhances gait stability and efficiency by re-establishing coordination among upper limb motion, trunk movement, and lower limb motion during walking. Notably, the concurrent reductions in RMS and RMS* and improvements in basic gait parameters indicate that improvements in trunk control and gait efficiency progressed in parallel, suggesting that recovery of shoulder function may promote neuromuscular integration of gait control. However, established minimal clinically important differences for trunk acceleration indices such as RMS and RMS* have not been clearly defined for older populations. Although the observed changes were statistically significant, the clinical impact of these improvements should be interpreted with caution.

Improvements in walking speed are closely associated with prognosis in older adults. Studenski et al. reported that survival increases with every 0.1 m/s increment in walking speed^15^. Therefore, improvements in gait stability and speed demonstrated in this study may contribute not only to reduced fall risk but also to improved life expectancy.

Furthermore, previous findings that RTSA for proximal humeral fractures significantly reduces 1-year mortality compared with conservative treatment are consistent with our results, suggesting that functional recovery achieved by RTSA favorably influences systemic health outcomes^16^.

From a clinical perspective, postoperative rehabilitation following RTSA should incorporate trunk and balance training aimed at early recovery of gait stability, in addition to pain management and range-of-motion exercises. In particular, the significant decrease in velocity-corrected RMS* observed in this study suggests that improvements in trunk stability beyond speed dependency may be achievable, underscoring the value of early low-load gait training and posture-control exercises after surgery. Moreover, trunk sway indices such as RMS may serve as objective tools for evaluating gait stability. However, established normative reference ranges for trunk acceleration indices in older populations have not been clearly defined.

This study has some limitations. First, this was a single-arm observational study without a control group, limiting causal inference. General postoperative recovery, including mobilization and pain reduction, may also have contributed to the observed gait improvements. Second, the sample size was small, and all surgeries were performed by a single surgeon at a single institution; therefore, selection bias cannot be entirely excluded. Missing data at some time points may have reduced statistical power. Third, although accelerometry was used for gait analysis, electromyography and three-dimensional motion analysis were not used; therefore, direct evaluation of the coordination among upper limb swing, trunk rotation, and lower limb motion was not possible, and changes in neuromuscular control mechanisms could not be directly demonstrated. Fourth, pain, shoulder range of motion, activities of daily living, and quality-of-life outcomes were not assessed concurrently. Fifth, the observation period was limited to 3 months; therefore, longer-term gait adaptation was not evaluated. Longer-term multicenter studies integrating subjective and objective outcomes are warranted.

In conclusion, after RTSA, trunk acceleration variability (RMS and RMS*) decreased and gait efficiency improved, with changes emerging by 1 month in normal walking and by 3 months in maximum walking. These results suggest that RTSA may contribute to improved whole-body gait control in addition to restoring shoulder function.
